# Comparative Evaluation of the Texture, Taste, and Flavor of Different Varieties of White Radish: Relationship Between Substance Composition and Quality

**DOI:** 10.3390/foods15010103

**Published:** 2025-12-29

**Authors:** Xinzhu Cai, Wanfu Hou, Li Zhang, Qingbiao Wang, Tianran Liu, Xiaoyan Zhao, Dan Wang

**Affiliations:** 1 College of Food Science, Shenyang Agricultural University, Shenyang 110866, China; 2 Institute of Agri-Food Processing and Nutrition, Beijing Academy of Agriculture and Forestry Sciences, Beijing 100097, China; 3 Beijing Key Laboratory of Fruits and Vegetables Preservation and Processing, Key Laboratory of Vegetable Postharvest Processing, Ministry of Agriculture and Rural Affairs, Beijing 100097, China; 4 Beijing Vegetable Research Center, Beijing Academy of Agriculture and Forestry Sciences, Beijing 100097, China

**Keywords:** white radish, cell wall materials, electronic nose, electronic tongue, principal component analysis

## Abstract

A systematic understanding of the overall flavor and taste characteristics across different white radish varieties is still lacking. This study selected six white radish varieties and analyzed their texture, taste, and flavor profiles. The results showed that JYHX had excellent hardness and chewiness, and CKJRM had the highest brittleness. The total sugar content of XY418 was the highest, and the sweetness was the most prominent. The umami and bitterness of CKXY and XY477 contributed significantly. A total of 43 volatile compounds were detected by gas chromatography–mass spectrometry (GC-MS), and CKFM12 had the highest content of sulfur-containing compounds. Dimethyl trisulfide and erucin were the key substances for the characteristic flavor of white radish. In this study, the texture, taste, and flavor characteristics of several white radish varieties and their potential biochemical components (cell wall substances, amino acids, volatile compounds) were comprehensively compared and analyzed for the first time. These findings provide a scientific basis for targeted quality evaluation, flavor improvement, and variety selection based on specific cooking applications and processing needs.

## 1. Introduction

Radish is an important rhizome vegetable in China and has a long history of planting and eating [1]. It is rich in nutrients, including vitamin C, dietary fiber, minerals, isothiocyanates, and other bioactive components. It also has the medicinal value of clearing heat and detoxifying, invigorating the spleen, and eliminating food, which is deeply loved by consumers [2,3]. According to the characteristics of skin color and root shape, radish can be divided into several types, and there are significant differences in color, spiciness, and nutritional components among different varieties [4]. Among them, white radish has become the most widely consumed variety due to its high yield and strong adaptability. However, there are also some differences in key quality characteristics (such as texture, flavor, and nutritional composition) among different varieties. The differences in genes and enzymes among different varieties can lead to changes in the content of erucin and isothiocyanate [5,6,7,8,9]. Furthermore, these quality variations may arise from distinct metabolic regulation between amino acid metabolism and volatile formation, as well as from the modulation of glucosinolate hydrolysis pathways by environmental factors such as sulfur nutrition and light conditions [10,11,12,13].

The texture, taste, and flavor of fruits and vegetables are the key factors determining their quality. Texture refers to the physical properties perceived during mastication, such as hardness, brittleness, and chewiness, which directly influence the consumer’s eating experience [14]. In white radish, crisp and juicy texture is more popular, while hard texture or high fiber content may reduce its commodity value. Studies indicate that texture characteristics are closely associated with cell structure, as well as pectin and cellulose content. Among them, the content of cellulose and hemicellulose is the key factor to determine its hardness and brittleness [15]. Non-volatile components (sugars, amino acids) interact with volatile compounds (such as aldehydes, alcohols, esters) to give white radish a unique taste and flavor [16]. Flavor is mainly composed of volatile organic compounds, and its type and content vary with variety, growth environment, and maturity [17]. Different white radish varieties have significant differences in texture and flavor, which affect their processing and application. For example, some varieties are suitable for fresh food, while others are suitable for processing into pickled or dried products.

However, systematic comparative studies on texture, taste, and flavor across different white radish varieties remain limited. This gap limits the overall understanding of variety differences and their applicability to specific consumption or processing uses. Therefore, this study used a texture analyzer, an electronic tongue, an electronic nose, and GC-MS to quantitatively analyze the texture and flavor components of different white radish varieties. The aim was to systematically analyze the texture and flavor characteristics of each variety, reveal its internal differences, and provide a scientific basis for variety breeding, quality control, and processing applications.

## 2. Materials and Methods

### 2.1. Chemicals

The enzyme-linked immunosorbent assay (ELISA) kits (KIRbio, Beijing Jinzhiyan Biotechnology Co., Ltd., Beijing, China) were used for the determination of vitamin C, total sugar, cell wall-related substances, and related enzyme activities. 3-nonanone as internal standard, purity ≥ 99.5% was provided by Shanghai Aladdin Biochemical Technology Co., Ltd. (Shanghai, China). Concentration of hydrochloric acid ≥ 36%, phenol, and sodium hydroxide, all from Sigma-Aldrich (St. Louis, MO, USA).

### 2.2. Sample and Treatment

Six different white radish varieties were collected from the Bashang Base of Vegetable Research Center of Beijing Academy of Agriculture and Forestry Sciences, including CKJRM, JYHX, CKFM12, XY418, CKXY, and XY477. All radishes were planted under the same agricultural conditions (consistent soil type, fertilization scheme, irrigation system) and harvested when they reached commercial maturity in the same growing season, in accordance with the Chinese national standard for radish quality (NY/T 1267-2007 Radishes [18]). After harvesting, select individuals with regular shapes, no pests or diseases, and uniform sizes for subsequent experiments. Samples were immediately transported to the laboratory and temporarily stored at 0 °C. Before analysis, the radishes were washed, peeled, and the edible central part of the rhizome was cut. To meet the different requirements of subsequent experimental procedures, some tissues were used for real-time analysis of fresh samples, while the remaining tissues were frozen in liquid nitrogen and stored at −80 °C for analysis.

### 2.3. Determination of Basic Indicators

Moisture content was measured with a rapid moisture analyzer (HS153, Mettler Toledo, Shanghai, China). Approximately 1 g of fresh radish tissue was placed on the sample tray and analyzed using the K105-3 measurement program. For pH determination, 20 g of the edible portion (washed and peeled) was chopped and homogenized for 30 s. The resulting slurry was then subjected to pH measurement using a calibrated pH meter (SevenCompact S210, Mettler Toledo, Shanghai, China). To determine Brix degrees, another 20 g of similarly prepared tissue was homogenized, and the juice was extracted through double-layer gauze. The filtered juice was analyzed with a refractometer (PAL-1, ATAGO, Tokyo, Japan) [19], and the stabilized reading was recorded as the percentage Brix. Vitamin C content was quantified using a commercial double-antibody sandwich ELISA kit according to the manufacturer’s protocol. All measurements were performed in triplicate, and mean values were calculated.

### 2.4. Texture Analysis

A texture analyzer (TA. XT Plus, Stable Micro Systems, Godalming, UK) was used to assess the hardness, brittleness, and chewiness of the six white radish varieties. Uniform 5 mm thick slices were prepared from the radishes, and puncture tests were performed with a cylindrical P/5 probe (5 mm diameter). Before the start of the test, the force and distance of the texture analyzer were calibrated using standard weights and built-in calibration procedures. The instrument parameters were set as follows: correction height, 10 mm; puncture distance, 9 mm; test speed, 0.4 mm/s; and post-test speed, 10 mm/s. To ensure data representativeness, three puncture tests were conducted at distinct positions (>5 mm from the edge) per slice, with three slices analyzed per variety (totaling nine replicates per variety) [20].

### 2.5. Analysis of Pectin and Cellulose Substances Affecting Texture

In this study, the related substances in the cell wall of white radish were determined, including protopectin, covalently bound pectin (CSP), ion-bound pectin (ISP), water-soluble pectin (WSP), and galacturonic acid. All parameters were measured using corresponding assay kits, with results expressed as mg/g. The original pectin was hydrolyzed in dilute acid, and the product was condensed with carbazole. Based on the absorbance at 530 nm, the content was determined. CSP and ISP were determined by sulfuric acid-carbazole colorimetry. After extraction with a specific extractant, the absorbance at 530 nm was measured by a spectrophotometer, and the standard curve was drawn, and the content was calculated. The content of WSP was determined by carbazole colorimetry after extraction with an acid solution. The content of galacturonic acid was determined by an ELISA kit. The absorbance at 450 nm wavelength was measured by the double antibody sandwich method, and the sample concentration was calculated.

In this study, ELISA was employed to quantify the activities of hemicellulose, cellulose, polygalacturonase, pectin methylesterase, and cellulase. All kits were based on the principle of the double antibody one-step sandwich method. After binding to the target molecule by horseradish peroxidase-labeled detection antibody, the absorbance (OD value) was measured at 450 nm wavelength by TMB substrate color reaction to calculate the sample concentration.

### 2.6. Electronic Tongue Analysis

The electronic tongue (INSENT, Inc., Atsugi-chi, Japan) system contained six taste sensor probes: CAO (sour), COO (bitter), AE1 (astringent), AAE (umami), CTO (salty), and GL1 (sweetness), and was also used to analyze bitter aftertaste, astringent aftertaste, and umami aftertaste (richness). Fresh middle pulp tissue was collected after peeling. A total of 30 g of sample was weighed, added with 120 mL of deionized water, and mixed for the beating treatment. The beating sample was placed in a centrifuge and centrifuged at 5000 rpm for 15 min at 10 °C. After centrifugation, gauze filtration was used to collect 100 mL of supernatant for electronic tongue analysis. A standard reference solution is used for calibration to ensure the accuracy of the sensor response. All measurements were performed in triplicate and reported as mean values [21].

### 2.7. Total Sugar, Total Acid

The total sugar content was determined by an ELISA kit. The specific steps were as follows: The edible part (the middle part of the rhizome) was cut, immediately frozen with liquid nitrogen, ground into powder, and analyzed. Using a double antibody one-step sandwich ELISA kit, the standard and sample were added to the pre-coated micropores, and horseradish peroxidase-labeled detection antibodies were added in turn. After incubation and washing, the TMB substrate was used for color development, and the absorbance (OD value) was measured at a wavelength of 450 nm. The total sugar concentration in the sample was calculated using the standard curve equation. The method is accurate and sensitive, and provides reliable data support for subsequent research.

The edible part (middle part of rhizome) was cut for the analysis of total acid, and three repeated experiments were carried out in strict accordance with the Chinese national standard (GB 12456-2021 [22]).

### 2.8. Amino Acids

In this study, the amino acid content of radish samples was determined using a Hitachi High Speed Amino Acid Analyzer (L-8900, Tokyo, Japan) by post-column derivatization ion-exchange chromatography with ninhydrin as specified in the National Standard for Food Safety Determination of Amino Acids in Food (GB 5009.124-2016 [23]). The specific steps were as follows: The edible part (the middle part of the rhizome) was cut and immediately frozen in liquid nitrogen. After the frozen samples were crushed, a certain amount of the sample was accurately weighed in the hydrolysis tube, and 6 mol/L hydrochloric acid solution and a small amount of phenol were added. After freezing, vacuuming, and nitrogen filling, the samples were hydrolyzed in a thermostat at 110 °C ± 1 °C for 22 h. The hydrolysates were treated by filtration, constant volume, vacuum drying, dissolution, and filtration to obtain the determination solution. The content of each amino acid was calculated by the external standard method with a mixed amino acid standard working solution as control [24].

### 2.9. Electronic Nose Analysis

An electronic nose (PEN 3, Airense Analytics GmbH, Schwerin, Germany) was used to analyze the flavor of white radish. It included 10 metal sensors-W1C (sensitive to aromatic compounds), W5S (sensitive to nitrogen oxides), W5C (sensitive to short-chain alkanes aromatic compounds), W3C (sensitive to ammonia and aromatic compounds), W6S (sensitive to hydrides), W1S (sensitive to methyl compounds), W1W (sensitive to sulfides), W2S (sensitive to alcohols, aldehydes and ketones), W2W (sensitive to aromatic components and organic sulfides) and W3S (sensitive to long-chain alkanes). The fresh middle pulp tissue after peeling was cut into uniform small pieces for direct analysis. A 3 g sample was placed into a 30 mL headspace vial and allowed to equilibrate for 30 min at room temperature. Then the samples were analyzed by an electronic nose with the headspace inspiratory method. Before sample analysis, clean air was used for calibration to ensure the accuracy of the sensor response. The measurement was performed using the following parameters: sensor cleaning time 180 s, zeroing time 5 s, sample preparation time 5 s, injection flow rate 300 mL/min, and detection time 180 s. After each test, the system was cleared and standardized, and then the next headspace sampling was performed, followed by data extraction and analysis at 175–177 s [25].

### 2.10. Headspace Gas Solid-Phase Microextraction (HS-SPME) and Gas Chromatography–Mass Spectrometry (GC-MS)

The volatile flavor compounds of different varieties of white radish were analyzed by GC-MS (Agilent 7890B, Santa Clara, CA, USA). The edible part (the middle part of the rhizome) was cut, immediately frozen with liquid nitrogen, ground into powder, and analyzed. Before the start of the analysis sequence, the GC-MS system was tuned, and the quality accuracy was calibrated. Headspace solid phase microextraction: 2.5 g of sample was weighed directly into a 15 mL headspace vial and sealed. The extraction conditions were in accordance with the previous optimized conditions of the laboratory; the sample vial was equilibrated at 45 °C for 30 min in a constant temperature water bath, and then the needle of the SPME manual injection handle was inserted into the injection bottle. The volatile compounds were extracted using a DVB/CWR/PDMS SPME fiber for 30 min, and then retracted. Then the extraction head was inserted into the gas chromatograph inlet, and the fiber head was extended for 5 min. The fiber was then retracted, and the SPME device was removed for immediate introduction into the GC-MS injector. After the fiber was retracted, the extraction head was taken out and analyzed.

The chromatographic column was HP-5MS UI (30 m × 0.25 mm, 0.25 μm), an elastic quartz capillary column. The column temperature programmed heating method was optimized: The temperature was programmed as follows: 36 °C (hold 3 min), then to 65 °C at 5 °C/min, to 108 °C at 3 °C/min, to 110 °C at 2 °C/min, to 155 °C at 3 °C/min, and finally to 200 °C (hold 3 min) at 5 °C/min. No diversion; carrier gas He, flow rate 1.2 mL/min; the extraction head was desorbed at 250 °C for 5 min at the inlet. The auxiliary heater temperature was 280 °C [26].

Mass spectrometry conditions: electron bombardment ion source (EI), electron energy of 70 eV, with ion source and interface temperatures set at 230 °C and 280 °C, respectively, and a mass scan range of *m*/*z* 50–550 amu.

Quantitative analysis: Quantification was performed by the internal standard method, and 3-nonanone was selected as the internal standard. The concentration was 0.4 g/L, and the addition amount was 3 μL. The relative content of each analyte was determined by its peak area relative to that of the internal standard [27]. The calculation formula was as follows: the content of each component (μg/g) = the peak area of each component/the peak area of internal standard × the concentration of internal standard (μg/mL) × 3 μL/sample volume (g) [28].

### 2.11. Statistical Analysis

Each experiment was repeated three times independently. Based on the radar chart, principal component analysis and correlation analysis were carried out using Origin 2024 (OriginLab, Northampton, MA, USA). Analysis of variance (ANOVA) followed by Duncan’s multiple-range test was performed with SPSS Statistics 27 (IBM, Chicago, IL, USA), setting the significance levels (*p* < 0.05).

## 3. Results and Discussion

### 3.1. Basic Indicator Analysis

**Figure 1:**
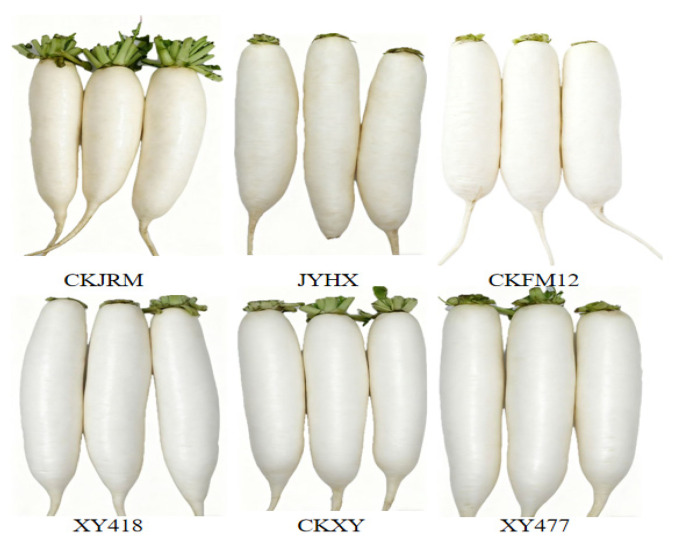
Six different varieties of white radish were CKJRM, JYHX, CKFM12, XY418, CKXY, XY477.

Figure 1 intuitively shows the appearance characteristics of the six white radish varieties. Although they are similar in appearance, they show significant differences in intrinsic quality, such as texture.

Based on the data in Table 1, there were significant differences in the basic indicators of the six white radish varieties. Among them, XY418 had the highest moisture content (92.36%) and the highest pH value (5.73). The water content of CKFM12 was the lowest (89.96%), but the brix degree was the highest (5.13%), suggesting that its sugar accumulation was more prominent. In terms of vitamin C content, JYHX had the highest content (0.87 mg/g), which had good nutritional potential. XY418 had the lowest vitamin C content (0.39 mg/g). These differences in basic physical and chemical properties provide an important basis for understanding the diversity of various varieties in texture, taste, and flavor.

### 3.2. Analysis of Texture Characteristics

#### 3.2.1. Texture

**Figure 2:**
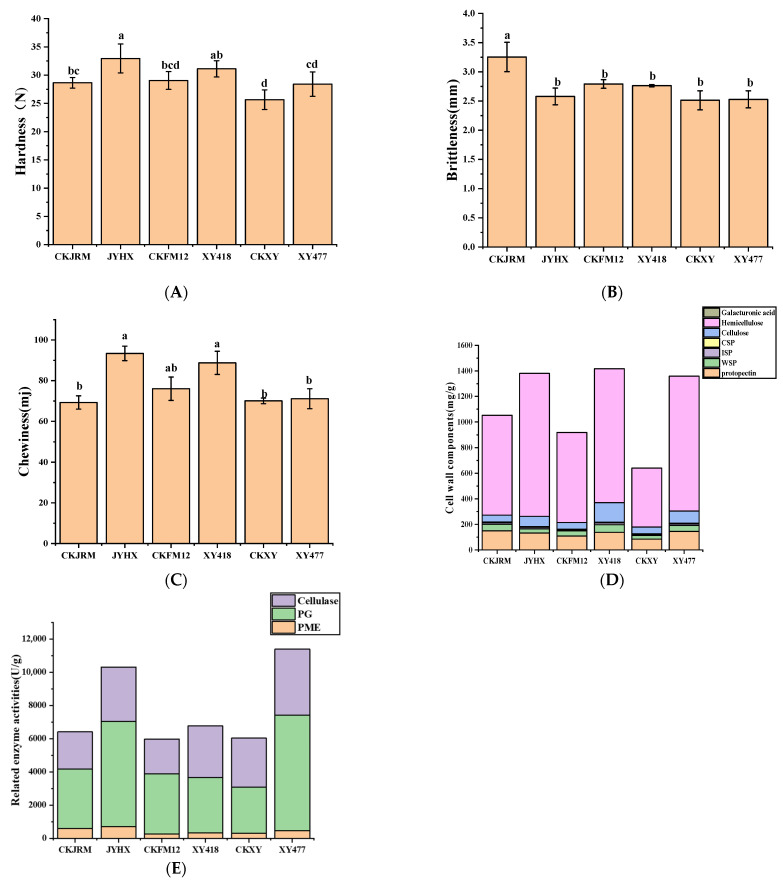
Hardness (**A**), brittleness (**B**), chewiness (**C**), the content of cell wall components (**D**), and related enzyme activities (**E**) of different varieties of white radish. Different lowercase letters (a to d, *p* < 0.05) indicate statistical differences between different varieties.

In this study, the texture characteristics of six varieties of white radish were systematically evaluated, including hardness, brittleness, and chewiness [29]. As shown in Figure 2A–C, six varieties of white radish showed significant differences in hardness, brittleness, and chewiness (*p* < 0.05). Chewiness analysis showed that JYHX and XY418 had higher chewiness, which were 93 mJ and 89 mJ, respectively, while CKJRM had the lowest chewiness of 70 mJ [30]. Previous studies have shown that the changes in texture characteristics during the growth of sweet potato are closely related to cell wall composition and related enzyme activities [31]. It is speculated that the differences in texture characteristics of different white radish varieties may be related to the changes in pectin, cellulose, and other components in the cell wall and their related enzyme activities, which are further verified by cell wall composition analysis.

#### 3.2.2. Analysis of Cell Wall Substances

To deeply analyze the relationship between the texture characteristics of white radish and cell wall substances, the cell wall components of six varieties were determined in this study, including pectin (protopectin, water-soluble pectin, ion-bound pectin, covalently bound pectin), cellulose (cellulose, hemicellulose) and galacturonic acid (Figure 2D), and the key enzyme activities related to cell wall degradation were analyzed, such as pectin methylesterase, polygalacturonase and cellulase (Figure 2E).

Differences in cell wall substance content were observed between the white radish varieties. Among them, the hemicellulose and ion-bound pectin content of the CKXY variety was the lowest, which was 461.34 mg/g and 8.73 mg/g, respectively. The hemicellulose content of JYHX was the highest (1117.64 mg/g), and its ion-binding pectin content also showed a higher trend (12.33 mg/g). In terms of enzyme activity, the cellulase activity of CKJRM was 2238.57 U/g; the cellulase activity of CKXY and XY477 was higher, reaching 2953.90 U/g and 3983.38 U/g, respectively. The observed texture differences among varieties can be mechanistically linked to their cell wall composition and enzyme activities. Varieties with higher ion-bound pectin and hemicellulose content (e.g., JYHX) exhibited greater hardness, likely due to enhanced cell wall rigidity and cross-linking, as similarly reported in calcium-treated radish and citrus peel [32,33]. Conversely, higher cellulase activity in varieties such as CKXY and XY477 was associated with reduced brittleness, suggesting that enzymatic degradation of cellulose may compromise structural integrity, a phenomenon also observed in Huanghua pear [34]. These findings highlight the role of cell wall metabolism in determining textural properties of white radish. Pectin methylesterase catalyzes the deesterification of pectin, generating free carboxyl groups. These groups can form strong ‘egg-box’ structures through cross-linking with calcium ions, thereby increasing tissue hardness [35,36,37]. In terms of chewiness, JYHX and XY418 varieties not only had higher chewiness, but also had relatively higher cellulose and hemicellulose contents. On the contrary, CKJRM and CKXY varieties with lower chewiness showed lower cellulose and hemicellulose content. It was further illustrated that the content of cellulose and hemicellulose had a certain effect on chewiness [31].

#### 3.2.3. Correlation Analysis

**Figure 3:**
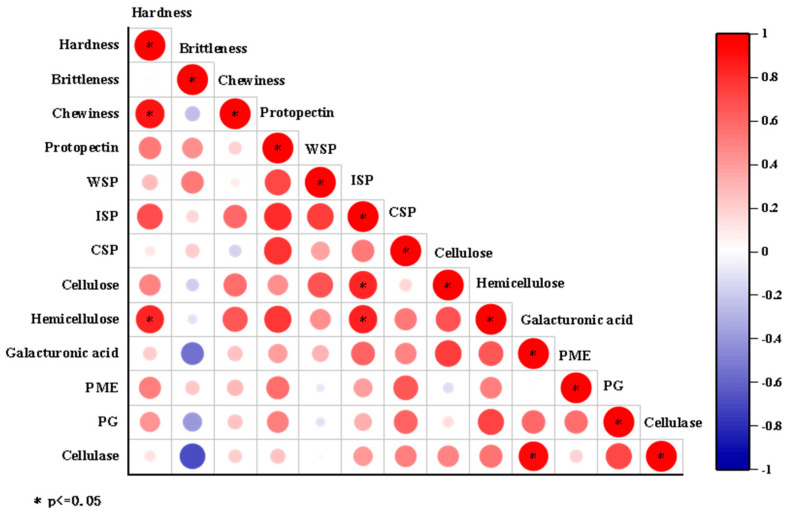
Correlation analysis between cell wall composition and texture characteristics of different varieties of white radish.

In this study, the correlation between texture characteristics, cell wall components, and cell wall-degrading enzyme activity was analyzed by correlation heat map (Figure 3). Based on confirming that there were significant differences in texture characteristics and cell wall material content among different white radish varieties, the intrinsic relationship between texture indexes and cell wall components and enzyme activities was further explored. The results of correlation analysis showed that there was a significant positive correlation between hardness and various pectin components (especially protopectin and ion-bound pectin) and hemicellulose content (*p* < 0.05). These results suggest that pectin and cellulose content may play an important role in the hardness of white radish tissue. Brittleness was positively correlated with protopectin and water-soluble pectin. There was a strong negative correlation with galacturonic acid, polygalacturonase, and cellulase, indicating that the increase in cell wall degradation may weaken tissue brittleness. In addition, chewiness was correlated with several cell wall components and enzyme activities; it was positively correlated with cellulose and hemicellulose content. This implies that cellulose abundance in the cell wall may be associated with chewing resistance in white radish.

### 3.3. Taste Analysis

#### 3.3.1. Electronic Tongue

**Figure 4:**
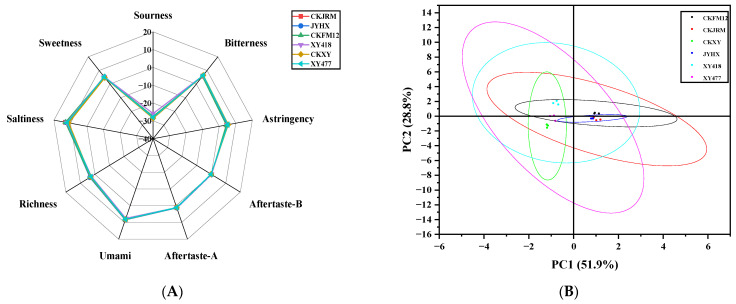
Electronic tongue radar (**A**) and PCA of different varieties of radish (**B**).

The differences in taste characteristics of different varieties of white radish were visually displayed by a radar map (Figure 4A). Overall, the six varieties exhibited higher umami and saltiness values, but lower sourness values. The sour response values of different white radish varieties were quite different. Principal component analysis (PCA) was further used to reduce the dimensionality of electronic tongue data. The first two principal components (PC1 and PC2) explained 80.7% of the cumulative variance (Figure 4B), indicating that these two principal components could reveal most of the information of the original data. The PCA diagram successfully distinguished six varieties, indicating that there were differences in their taste characteristics. In the PCA diagram, the distribution positions of CKFM12, CKJRM, and JYHX were close, indicating that their overall taste characteristics were similar. Previous studies have reported that the electronic tongue response is influenced by free amino acid content [38]. Therefore, it is speculated that the difference in taste of different varieties of white radish may be related to its total sugar, total acid content, and amino acid composition, which will be verified by further analysis.

#### 3.3.2. Total Sugar and Total Acid

**Figure 5:**
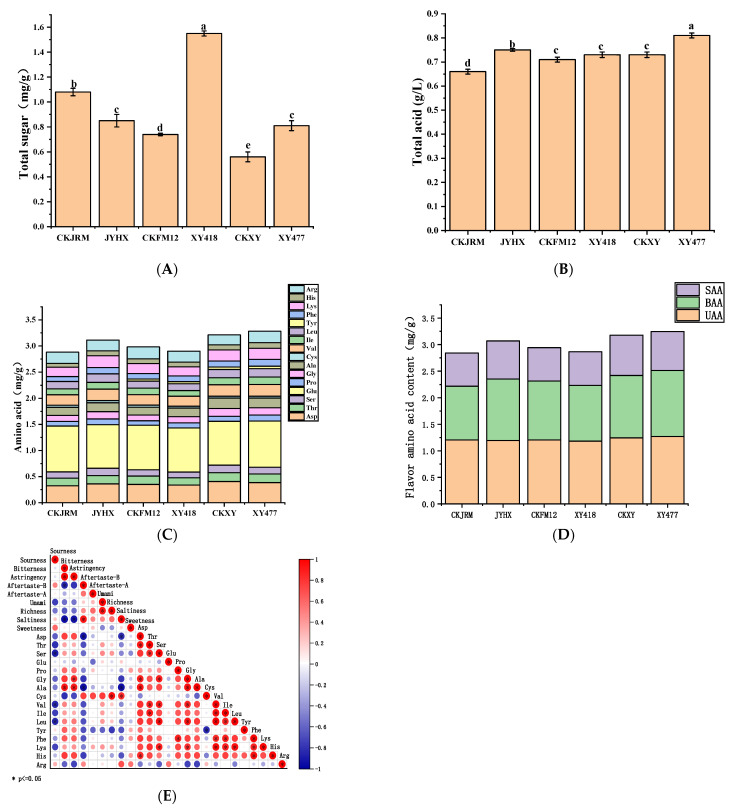
Total sugar content (**A**) and total acid content (**B**), amino acid content (**C**), flavor amino acid content (**D**), and correlation analysis between amino acid and electronic tongue response value (**E**) of different varieties of white radish. Different lowercase letters (a to e, *p* < 0.05) indicate statistical differences between different varieties.

The determination results of the total sugar content of six white radish varieties are shown in Figure 5A, and the range is 0.56–1.55 mg/g. Among them, the total sugar content of XY418 was the highest, reaching 1.55 mg/g, which was consistent with its highest sweet response value in electronic tongue analysis. In contrast, the total sugar content of CKXY was the lowest, only 0.56 mg/g, and its sweetness value was the lowest. The above results showed that there was a positive correlation between the sweetness value and the total sugar content of white radish. In addition, there were also differences in total acid content among different white radish varieties, as shown in Figure 5B. Among them, XY477 and CKJRM had the highest and lowest total acid content, respectively. Combined with the analysis of electronic tongue data, the sourresponse values of CKXY, JYHX, and XY477 were relatively high, which were in agreement with the measured total acid content.

#### 3.3.3. Amino Acids

Amino acids serve not only as protein building blocks but also as key flavor compounds, contributing sweet, bitter, and umami tastes [39]. According to the taste characteristics, amino acids were categorized as bitter amino acids (BAA: Arg, Val, Phe, Ile, Leu, Lys, Tyr, His, Met), sweet amino acids (SAA: Pro, Ala, Ser, Gly, Thr), and umami amino acids (UAA: Asp, Glu) [40]. Sixteen amino acids were detected across the six white radish varieties (Figure 5C). XY477 exhibited the highest total amino acid content (3.3 mg/g), while XY418 showed the lowest (2.9 mg/g). Further analysis of the composition of flavor amino acids (Figure 5D) showed that the six varieties were rich in aspartic acid (Asp), glutamic acid (Glu), and arginine (Arg). These three amino acids are not only important components of protein, but also have important taste contributions. Aspartic acid and glutamic acid are important sources of umami, while arginine contributes to bitterness [41]. The content of aspartic acid varied greatly among different varieties, among which CKXY was the highest (0.405 mg/g) and CKJRM was the lowest (0.325 mg/g). In addition, CKXY exhibited the highest glutamic acid content (0.885 mg/g) among all varieties, which further enhanced its umami intensity. Combined with the electronic tongue data, it was found that the sweet response values of CKFM12 and CKJRM were low, which was consistent with the low sweet amino acid content. The bitterness response values of XY477 and CKXY were higher, which was consistent with the high content of bitter amino acids. In addition to the above main flavor amino acids, the contents of other essential amino acids, such as phenylalanine (Phe), valine (Val), and lysine (Lys), are also considerable. These amino acids may have a comprehensive effect on the overall taste characteristics of white radish.

In order to further analyze the contribution of amino acid composition to the overall taste characteristics of white radish, this study further analyzed the correlation of taste-related substances in six radish varieties (Figure 5E). The results showed that the content of umami amino acids (aspartic acid and glutamic acid) was positively correlated with the umami response value of electronic tongue, indicating that the two were the key flavor substances that determined the umami intensity of radish. There was also a positive correlation between sweet amino acids (such as proline) and sweet response values, which further confirmed the synergistic enhancement of sweet amino acids on overall sweetness. In addition, the content of bitter amino acids (such as phenylalanine and histidine) was positively correlated with the bitterness response value, indicating that the accumulation of bitter amino acids was the main factor causing the bitterness difference between varieties. In summary, there is a clear correspondence between the taste profile of electronic tongue and its characteristic amino acid composition in different white radish varieties.

### 3.4. Flavor Analysis

#### 3.4.1. Electronic Nose

**Figure 6:**
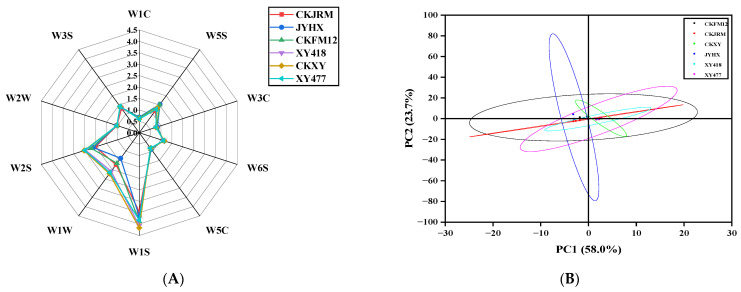
Electronic nose radar (**A**) and PCA of different varieties of radish (**B**).

Electronic nose analysis is widely used in food research to distinguish overall flavor characteristics [4]. In this study, the overall flavor of six varieties of white radish was analyzed by electronic nose (Figure 6A). The results showed that the response values of W1S, W1W, and W2S sensors varied greatly among different varieties, indicating that the distribution of methyl, sulfide, alcohol, and aldehyde ketone compounds had obvious variety specificity. The response value of CKXY was higher, while that of JYHX was lower. In addition, all varieties showed strong responses to these three sensors, indicating that the above-mentioned flavor substances were abundant; in contrast, the response of all white radish samples to W1C, W3C, and W5C sensors was generally weak, indicating that the content of ammonia, aromatic compounds, and alkanes was low.

On this basis, PCA was further carried out. The results are shown in Figure 6B. The positions of CKXY and XY477 in the figure are close, indicating that the flavor characteristics of the two are similar. The first two principal components (PC1 and PC2) explained 81.7% of the cumulative variance, which could clearly distinguish the six white radish varieties, indicating that there were significant differences in the overall flavor among the varieties.

#### 3.4.2. GC-MS

Six white radish varieties were analyzed by solid phase microextraction–gas chromatography–mass spectrometry (HS-SPME/GC-MS). A total of 43 volatile compounds were detected, including 9 isothiocyanates, 7 sulfides, 7 heterocycles, 4 esters, 3 aldehydes, 3 alkanes, 3 terpenes, 3 ketones, 2 alcohols, 1 ether, and 1 acid (Table S1). The results revealed distinct profiles in the types and contents of volatile compounds among the white radish varieties, which may be related to the genetic background and growth environment of white radish [42]. It has been pointed out that the main source of spicy flavor in white radish is the degradation products of glucosinolates, especially isothiocyanate compounds, which not only give white radish a unique pungent odor, but also have potential anticancer activity and nutritional value [43]. The results of this study further confirmed that isothiocyanates are the most important flavor substances in white radish. Among them, the highest content is erucin (from 1.179 μg/g of CKXY to 3.632 μg/g of CKFM12), which has a spicy taste and is a key component that constitutes the characteristic flavor of white radish. In addition, volatile components such as esters, alcohols, and ethers further enriched the complex flavor characteristics of white radish [44].

Among the detected volatile compounds, aldehydes were mainly nonanal and decanal, and ketones were represented by 3-Pentanone and 3,5-Heptanedione, 2,6-dimethyl-. It is worth noting that there are 22 identical volatile compounds in all varieties, and the varieties with the highest and lowest volatile compound content are XY418 (35) and CKXY (26). The total content of volatile compounds ranged from the lowest in CKXY to the highest in JYHX. Among them, the content of sulfide and heterocyclic substances in JYHX and CKFM12 was higher, which was consistent with the higher response value of this variety on the W2W sensor (sensitive to aromatic components and organic sulfides) in electronic nose analysis, indicating that its flavor was stronger.

**Figure 7:**
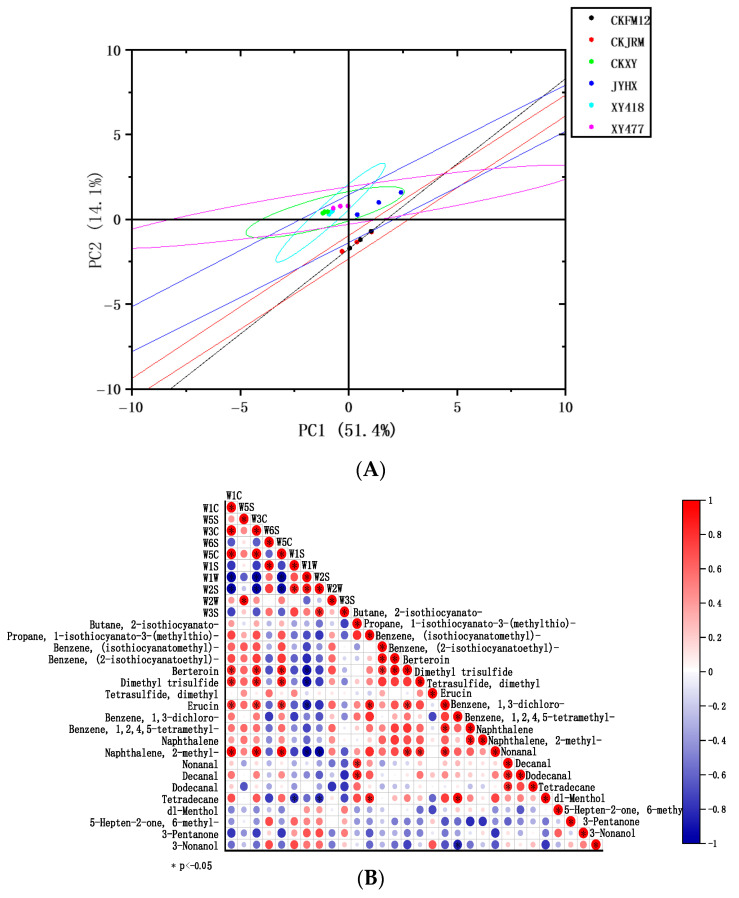
Principal component analysis of flavor in different varieties of white radish (**A**), correlation analysis heatmap between electronic nose sensor values and flavor compounds (**B**).

To discern inter-varietal differences in volatile compounds, PCA was performed on common flavor compounds to visualize aroma similarities and differences. As shown in Figure 7A, XY477, CKXY, and XY418 were similar in flavor characteristics, while JYHX was significantly different from other varieties.

The odor activity value (OAV) is analyzed. Odor activity value (OAV) comprehensively reflects the concentration of volatile compounds and their odor thresholds, and is a key indicator for evaluating flavor contribution [45]. Table 2 shows the odor activity values (OAVs) of 20 compounds with thresholds that can be found, indicating that these compounds have a major contribution to the flavor formation of white radish. In particular, the OAV was significantly greater than 1, and the concentration was much higher than the threshold; that is, the contribution to the flavor was very significant. Further analysis showed that the OAVs of dimethyl trisulfide, tetrasulfide, dimethyl, and erucin were extremely high, which were the key contributors to the characteristic flavor of white radish. In addition, the odor description of propane, 1-isothiocyanato-3-(methylthio)- and dimethyl trisulfide is roughly defined as sulfur odor, which together constitute an indispensable part of the spicy flavor of white radish. Different varieties of white radish showed obvious variety specificity in the OAV of key flavor substances. For example, the OAV of dimethyl trisulfide was the highest in JYHX, indicating that JYHX had a stronger sulfur odor. The OAV of erucin in CKFM12 was as high as 1135, which was much higher than that of other varieties, further confirming the outstanding performance of this variety in spicy flavor. The CKFM12 variety had the highest erucin content (3.632 μg/g) and odor activity value (1135), and the spicy flavor was prominent, which was suitable for curing processing. Its spicy flavor could form a unique flavor of pickled radish after fermentation. The content of dimethyl trisulfide (0.107 μg/g) and total volatile substances (7.079 μg/g) of the JYHX variety were the highest, and the sulfur and spicy flavors were rich. It was suitable for making spicy dried radish, and the flavor could be further enriched after dehydration and seasoning. The CKXY variety had the lowest volatile matter content (2.292 μg/g) and a light flavor, which was suitable for stewing soup and did not produce a strong irritating odor during heating.

#### 3.4.3. Correlation Analysis

To clarify the response characteristics of each sensor of the electronic nose to different volatile compounds, correlations between main volatile compounds (GC-MS) and electronic nose sensor responses were examined. The results are shown in Figure 7B. The heat map showed that the W2W sensor (sensitive to aromatic components and organic sulfides) had a strong positive correlation with a variety of isothiocyanates (such as propane, 1-isothiocyanato-3-(methylthio)-, erucin, benzene, (2-isothiocyanatoethyl)-, berteroin and sulfides (such as dimethyl trisulfide, tetrasulfide, dimethyl), indicating that these sensors can effectively reflect the content changes of spicy flavor substances in white radish. Thus, the electronic nose and GC-MS provided mutually corroborative data, effectively capturing the varietal differences in key flavor compounds [46].

### 3.5. Comprehensive Integration of Texture, Taste, and Flavor Profiles

Instrumental analyses of texture, taste, and flavor were integrated to provide a comprehensive understanding of the quality characteristics of the six white radish varieties. JYHX exhibits excellent textural properties, including high hardness and chewiness, which are closely related to the increase in hemicellulose and ion-bound pectin content. In contrast, XY418 had the highest total sugar content, consistent with its prominent sweetness. CKFM12 stands out for its high erucin content and strong spicy flavor. CKXY and XY477 were characterized by higher umami amino acid content and softer texture, showing obvious taste and texture characteristics. These comprehensive findings illustrate the complex interactions among cell wall composition, taste-active compounds, and volatile characteristics. Together, they provide a multidimensional framework for understanding the intrinsic quality differences among white radish varieties.

## 4. Conclusions

In this study, six different varieties of white radish were studied comprehensively and deeply for the first time, and their overall texture, taste, and flavor characteristics were systematically observed and analyzed. The results showed that JYHX had high hardness, good chewiness, and rich flavor. CKFM12 has the highest content of sulfur-containing compounds and a prominent spicy taste. It is speculated that it may be more suitable for the production of processed products that need to maintain certain toughness and prominent flavor, such as spicy dried radish and flavor-pickled radish. The total sugar content and sweetness of XY418 were the highest. CKJRM has excellent brittleness and elegant aroma, and it is speculated that it is more suitable as a fresh radish. XY477 and CKXY have a high amino acid content, a high umami value, and a soft texture. These two varieties may be more suitable for stewing or soup and can better release umami substances during heating. These processing suitability suggestions are primarily based on instrumental analyses and physicochemical indicators and require further validation through sensory evaluation and practical processing trials. This study provides a theoretical foundation for white radish breeding and targeted processing. It also confirms the complementarity and reliability of GC-MS and E-nose in flavor analysis, offering methodological guidance for rapid and objective flavor assessment. In the future, it is necessary to comprehensively evaluate the promotion value of varieties by combining agronomic traits and cost-effectiveness.

## Figures and Tables

**Table 1:** Basic characteristic indicators of six varieties of white radish.

	Moisture (%)	PH	Brix Degrees (%)	Vitamin C (mg/g)
CKJRM	92.05 ± 0.01 ^ab^	5.22 ± 0.02 ^d^	3.9 ± 0.1 ^e^	0.46 ± 0.01 ^d^
JYHX	92.02 ± 1.55 ^ab^	5.4 ± 0.01 ^b^	4.1 ± 0.2 ^de^	0.87 ± 0.02 ^a^
CKFM12	89.96 ± 0.34 ^b^	5.04 ± 0.01 ^f^	5.13 ± 0.06 ^a^	0.58 ± 0.02 ^b^
XY418	92.36 ± 0.09 ^a^	5.73 ± 0.02 ^a^	4.53 ± 0.06 ^b^	0.39 ± 0.03 ^e^
CKXY	90.49 ± 1.41 ^ab^	5.15 ± 0.01 ^e^	4.3 ± 0.1 ^cd^	0.53 ± 0.03 ^c^
XY477	90.36 ± 0.17 ^ab^	5.3 ± 0.01 ^c^	4.43 ± 0.12 ^bc^	0.58 ± 0.03 ^b^

Different lowercase letters (a to e, *p* < 0.05) indicate statistical differences between different varieties.

**Table 2:** OAV of volatile compounds in six varieties of white radish.

Compound Name	Odor Description	Threshold (mg/kg)	OAV of VOCs in the Sample					
			CKJRM	JYHX	CKFM12	XY418	CKXY	XY477
Butane, 2-isothiocyanato-	green	0.09	0.044	ND	0.044	0.022	ND	ND
Propane, 1-isothiocyanato-3-(methylthio)-	sulfurous	0.65	0.571	0.415	0.728	0.248	0.212	0.255
Benzene, (isothiocyanatomethyl)-	spicy, oily	0.007	ND	0.143	ND	ND	ND	ND
Benzene, (2-isothiocyanatoethyl)-	green	0.24	0.508	1.158	0.500	0.313	0.192	0.554
Berteroin	cabbage, radish	0.8	0.399	0.804	0.453	0.085	0.08	0.321
Dimethyl trisulfide	sulfureous	0.0001	820	1070	840	800	600	570
Tetrasulfide, dimethyl	garlic, meaty	0.00002	1800	2700	2250	2950	2550	1450
Erucin	cabbage, radish	0.0032	854.063	1018.438	1135.000	561.25	368.438	654.063
Benzene, 1,3-dichloro-	sweet	0.17	0.024	0.012	0.024	ND	ND	0.018
Benzene, 1,2,4,5-tetramethyl-	rancid, sweet	0.061	0.049	0.082	0.082	0.049	0.016	0.066
Naphthalene	Pungent, dry	0.006	0.5	0.833	0.667	0.667	0.333	0.667
Naphthalene, 2-methyl-	oily, aromatic	0.003	1.333	1.667	1.333	ND	ND	ND
Nonanal	rose, fatty	0.0011	15.455	8.182	12.727	15.455	6.364	9.091
Decanal	fatty	0.003	6.333	4	5.667	5.667	2.667	2
Dodecanal	soapy, waxy	0.0002	15	ND	ND	10	ND	ND
Tetradecane	mild, waxy	1	1.667	0.667	1.333	ND	ND	0.667
dl-Menthol	peppermint, cool, woody	0.13	ND	ND	ND	ND	ND	0.046
5-Hepten-2-one, 6-methyl-	green, apple, banana	0.068	ND	ND	ND	ND	0.029	ND
3-Pentanone	ethereal, acetone	0.06	0.067	0.067	0.067	0.167	0.083	0.167
3-Nonanol	spice, herbal, oily	0.07	ND	ND	ND	0.029	0.014	ND

The odor threshold (mg/kg) in water was obtained from a book called Compilations of Odor Threshold Values in Air, Water, and Other Media (Second Enlarged and Revised Edition). OAV: odor activity value, which is the ratio of concentration to odor threshold. ‘ND’ means not detected.

## Data Availability

The original contributions presented in this study are included in the article/Supplementary Materials. Further inquiries can be directed to the corresponding author.

## Supplementary Materials

The following supporting information can be downloaded at https://www.mdpi.com/article/10.3390/foods15010103/s1. Table S1: The composition of volatile compounds in six radish varieties was studied by HS-SPME/GC-MS.
