# Supplementary material for: Comparative Evaluation of the Texture, Taste, and Flavor of Different Varieties of White Radish: Relationship Between Substance Composition and Quality
Source: Foods. 2025 Dec 29;15(1):103. doi: 10.3390/foods15010103 (PMC12785676; doi:10.3390/foods15010103)
Supplement: Supplementary file 1 [file foods-15-00103-s001.zip › foods-4033856-supplementary.pdf]

Table S1 The composition of volatile compounds in six radish varieties was studied by HS-SPME/GC-MS.

NO.	Compound name	CAS	Compound content(ug/g)					
			CKJRM	JYHX	CKFM12	XY418	CKXY	XY477
	Isothiocyanate							
1	Butane, 2-isothiocyanato-	4426-79-3	0.004 ± 0.001^{a}	ND	0.004 ± 0.000^{a}	0.002 ± 0.000^{b}	ND	ND
2	4-Methylpentyl isothiocyanate	17608-07-0	0.234 ± 0.047^{ab}	0.362 ± 0.147^{a}	0.215 ± 0.073^{ab}	0.150 ± 0.011^{b}	0.077 ± 0.004^{b}	0.215 ± 0.048^{ab}
3	Hexane, 1-isothiocyanato-	4404-45-9	0.284 ± 0.058^{ab}	0.484 ± 0.202^{a}	0.266 ± 0.084^{ab}	0.129 ± 0.006^{b}	0.092 ± 0.004^{b}	0.218 ± 0.044^{b}
4	Heptane, 1-isothiocyanato-	4426-83-9	0.038 ± 0.004^{b}	0.096 ± 0.043^{a}	0.029 ± 0.009^{b}	0.018 ± 0.001^{b}	0.006 ± 0.000^{b}	0.039 ± 0.008^{b}
5	Cyclohexanemethyl isothiocyanate	52395-66-1	ND	ND	ND	0.004±0.000	ND	0.024±0.000
6	Propane, 1-isothiocyanato-3-(methylthio)-	505-79-3	0.371 ± 0.246^{ab}	0.270 ± 0.120^{ab}	0.473 ± 0.101^{a}	0.161 ± 0.001^{b}	0.138 ± 0.014^{b}	0.166 ± 0.031^{ab}
7	Benzene, (isothiocyanatomethyl)-	622-78-6	ND	0.001±0.000	ND	ND	ND	ND
8	Benzene, (2-isothiocyanatoethyl)-	2257-09-2	0.122 ± 0.018^{b}	0.278 ± 0.129^{a}	0.120 ± 0.020^{b}	0.075 ± 0.004^{b}	0.046 ± 0.005^{b}	0.133 ± 0.016^{b}
9	Berteroin	4430-42-6	0.319 ± 0.119^{ab}	0.643 ± 0.302^{a}	0.362 ± 0.058^{ab}	0.068±0.091b	0.064 ± 0.006^{b}	0.257 ± 0.033^{b}
Total content			1.372	2.134	1.469	0.607	0.423	1.052
	Sulfur compounds							
1	Dimethyl sulfone	67-71-0	0.065 ± 0.024^{a}	0.087 ± 0.035^{a}	0.064 ± 0.027^{a}	0.069 ± 0.011^{a}	0.067 ± 0.004^{a}	0.076 ± 0.013^{a}
2	Dimethyl trisulfide	3658-80-8	0.082 ± 0.035^{a}	0.107 ± 0.040^{a}	0.084 ± 0.032^{a}	0.080 ± 0.017^{a}	0.060 ± 0.017^{a}	0.057 ± 0.009^{a}
3	2H-Azepine-2-thione,	7203-96-5	0.042 ± 0.012^{ab}	0.049 ± 0.019^{a}	0.040 ± 0.015^{ab}	0.024 ± 0.000^{ab}	0.017 ± 0.001^{b}	0.028 ± 0.006^{ab}

	hexahydro-							
4	Tetrasulfide, dimethyl	5756-24-1	0.036±0.024^{a}	0.054±0.022^{a}	0.045±0.012^{a}	0.059±0.003^{a}	0.051±0.029^{a}	0.029±0.006^{a}
5	trans-Raphasatin	13028-50-7	0.989±0.453^{ab}	1.304±0.570^{a}	1.158±0.214^{ab}	0.566±0.035^{ab}	0.423±0.040^{b}	0.715±0.119^{ab}
6	Erucin	4430-36-8	2.733±1.941^{a}	3.259±2.586^{a}	3.632±0.670^{a}	1.769±0.132^{a}	1.179±0.003^{a}	2.093±0.324^{a}
7	Sulforaphane	4478-93-7	0.005±0.002^{ab}	0.006±0.001^{a}	0.006±0.001^{a}	0.003±0.001^{ab}	0.002±0.001^{b}	ND
Total content			3.952	4.866	5.029	2.57	1.799	2.998
	Heterocyclic							
1	Benzene, 1,3-dichloro-	541-73-1	0.004±0.000	0.002±0.000	0.004±0.000	ND	ND	0.003±0.000
2	3-Piperidinol, 1-ethyl-	13444-24-1	0.009±0.002^{a}	0.008±0.003^{a}	0.008±0.003^{a}	0.005±0.000^{ab}	0.003±0.000^{b}	0.004±0.001^{ab}
3	Benzene, 1,2,4,5-tetramethyl-	95-93-2	0.003±0.002^{ab}	0.005±0.000^{a}	0.005±0.001^{a}	0.003±0.000^{ab}	0.001±0.000^{b}	0.004±0.001^{ab}
4	Naphthalene	91-20-3	0.003±0.000^{a}	0.005±0.002^{a}	0.004±0.000^{a}	0.004±0.000^{a}	0.002±0.000^{a}	0.004±0.001^{a}
5	Thiazole, 2,4,5-trimethyl-	13623-11-5	0.012±0.011^{ab}	0.003±0.000^{b}	0.016±0.005^{a}	0.003±0.001^{b}	0.013±0.004ab	0.014±0.002^{ab}
6	Cyclopentene, 1-pentyl-	4291-98-9	ND	ND	ND	0.009±0.000	ND	ND
7	Naphthalene, 2-methyl-	91-57-6	0.004±0.000^{a}	0.005±0.003^{a}	0.004±0.000^{a}	ND	ND	ND
Total content			0.035	0.028	0.041	0.024	0.019	0.029
	esters							
1	Formic acid, octyl ester	112-32-3	0.006±0.000	0.002±0.000	ND	ND	ND	ND
2	2-Nonenoic acid, methyl ester	111-79-5	ND	ND	ND	0.004±0.000	ND	ND
3	3,6-Dodecadienoic acid, methyl ester	16106-01-7	0.007±0.000	ND	ND	0.007±0.000	ND	ND
4	Dodecanoic acid, ethyl ester	106-33-2	ND	ND	ND	0.002±0.000	ND	ND
Total			0.013	0.002	ND	0.013	ND	ND

content								
	aldehydes							
1	Nonanal	124-19-6	0.017±0.007[a]	0.009±0.002[ab]	0.014±0.003[ab]	0.017±0.000[a]	0.007±0.000[b]	0.010±0.000[ab]
2	Decanal	112-31-2	0.019±0.010[a]	0.012±0.002[a]	0.017±0.007[a]	0.017±0.002[a]	0.008±0.000[a]	0.006±0.001[a]
3	Dodecanal	112-54-9	0.003±0.000	ND	ND	0.002±0.000	ND	ND
Total content			0.039	0.021	0.031	0.036	0.015	0.016
	alkanes							
1	1,3-Dioxolane, 2-methyl-2-pentyl-	4352-95-8	0.008±0.006[a]	0.004±0.000[a]	0.008±0.006[a]	0.011±0.000[a]	0.007±0.000[a]	0.008±0.001[a]
2	Tetradecane	629-59-4	0.005±0.000[a]	0.002±0.000[c]	0.004±0.000[b]	ND	ND	0.002±0.000[c]
3	Tetradecane, 2,6,10-trimethyl-	14905-56-7	0.002±0.000	ND	ND	ND	ND	ND
Total content			0.015	0.006	0.012	0.011	0.007	0.01
	Terpenoids							
1	dl-Menthol	89-78-1	ND	ND	ND	ND	ND	0.006±0.000
2	Cyclohexanol, 5-methyl-2-(1-methylethyl)-, [1S-(1.alpha.,2.beta.,5.beta.)]-	23283-97-8	ND	ND	ND	0.002±0.000	ND	ND
3	5,9-Undecadien-2-one, 6,10-dimethyl-, (Z)-	3879-26-3	0.006±0.000[c]	0.009±0.005[bc]	0.007±0.000[c]	0.017±0.002[a]	0.015±0.004[ab]	0.012±0.000[abc]
Total content			0.006	0.009	0.007	0.019	0.015	0.018
	Ketones							
1	5-Hepten-2-one, 6-methyl-	110-93-0	ND	ND	ND	ND	0.002±0.000	ND

2	3-Pentanone	96-22-0	0.004±0.000[c]	0.004±0.000[c]	0.004±0.000[c]	0.010±0.001[a]	0.005±0.000[b]	0.010±0.000[a]
3	3,5-Heptanedione, 2,6-dimethyl-	18362-64-6	0.005±0.000[ab]	0.004±0.000[b]	0.005±0.000[ab]	0.010±0.001[a]	0.005±0.000[ab]	0.007±0.004[ab]
Total content			0.009	0.008	0.009	0.02	0.012	0.017
	Alcohols							
1	3-Nonanol	624-51-1	ND	ND	ND	0.002±0.000	0.001±0.000	ND
2	1-Decanol, 2-hexyl-	2425-77-6	ND	ND	ND	0.002±0.000	ND	ND
Total content			ND	ND	ND	0.004	0.001	ND
	ethers							
1	Heptane, 1,1'-oxybis-	629-64-1	0.006±0.000[a]	0.005±0.002[a]	ND	0.002±0.000[ab]	0.001±0.000[b]	0.003±0.000[ab]
Total content			0.006	0.005	ND	0.002	0.001	0.003
	Acids							
1	Oleic Acid	112-80-1	ND	ND	ND	0.002±0.000	ND	ND
Total content			ND	ND	ND	0.002	ND	ND
Total content of VOCs			5.447	7.079	6.598	3.308	2.292	4.143

ND means not detected. Different lowercase letters (a to c, $p<0.05$) indicate statistical differences between different varieties.
